# Dating stalagmites in mediterranean climates using annual trace element cycles

**DOI:** 10.1038/s41598-017-00474-4

**Published:** 2017-04-04

**Authors:** Gurinder Nagra, Pauline C. Treble, Martin S. Andersen, Petra Bajo, John Hellstrom, Andy Baker

**Affiliations:** 1 Connected Waters Initiative Research Centre, University of New South Wales, Sydney, NSW 2052 Australia; 2 Australian Nuclear Science and Technological Organization, Lucas Heights, NSW 2234 Australia; 3 School of Earth Sciences, University of Melbourne, Melbourne, VIC 3010 Australia

## Abstract

Speleothems may preserve geochemical information at annual resolution, preserving information about past hydrology, environment and climate. In this study, we advance information-extraction from speleothems in two ways. First, the limitations in dating modern stalagmites are overcome by refining a dating method that uses annual trace element cycles. It is shown that high-frequency variations in elements affected by prior calcite precipitation (PCP) can be used to date speleothems and yield an age within 2–4% chronological uncertainty of the actual age of the stalagmite. This is of particular relevance to mediterranean regions that display strong seasonal controls on PCP, due to seasonal variability in water availability and cave-air *p*CO_2_. Second, using the chronology for one stalagmite sample, trace elements and growth-rate are compared with a record of climate and local environmental change i.e. land-use and fire, over the 20^th^ century. Well-defined peaks in soil-derived trace elements and simultaneous decreases in growth-rate coincide with extreme annual rainfall totals in 1934 and 1974. One of which, 1934, was due to a recorded cyclone. We also find that bedrock-derived elements that are dominated by PCP processes, reflect a well-known period of drying in southwest Australia which began in the 1970’s.

## Introduction

Stalagmites are invaluable geochemical archives of past climate^1–3^. However, the sensitivity of stalagmite geochemistry to local biomass, soil and karst processes^4–7^ suggests the need to validate the stalagmite record, using cave monitoring and modern stalagmites. Cave monitoring allows us to determine the sensitivity of dripwater hydrochemistry by comparing it with; rainfall, temperature, cave *p*CO_2_ and local environmental data, to determine the sensitivity of cave dripwaters to these variables^8^. Similarly, modern stalagmites also allow us to compare the stalagmite geochemical record to instrumental climate and local environmental data^9–11^. This deepens our knowledge of how dripwater geochemical signals are incorporated into the speleothem calcite.

Close comparison of the modern (i.e. 20^th^ century) stalagmite record with historical data, however, requires accurate and precise age constraints. U-Th or radiocarbon dating methods–key methods which are currently being used - have significant analytical limitations in modern speleothems. For U-Th dating, there is typically insufficient ingrowth of the daughter nuclide ^230^Th which causes large analytical errors. Precise U-Th ages are often also confounded by small amounts of detrital Th^12^. For radiocarbon dating, the elevated atmospheric ^14^C during the period of nuclear weapons testing may be detected in the speleothem (known as the ‘bomb spike’), to constrain post-1950 dates^13^, but this involves assumptions regarding lags in the soil C pool and the contribution of ‘dead’ C from the limestone or radiocarbon decayed C from soil organic matter. The pre-1950 ^14^C measurements are similarly limited^12^. These approaches may therefore result in significant chronological uncertainty for modern speleothems, hampering a direct comparison with historical data.

Annual lamina counting is an established method in tree-rings, ice-cores, corals, lake sediments and speleothems to reconstruct annually resolved records^1^. It may result in a more precise chronology if annual growth increments are resolvable and provided that there are no interruptions to growth in the record. In speleothems, these annual layers can be obtained from visible growth intervals^10^, fluorescent organic matter layers^1^, seasonal trace element cycles^14–19^ or O and C isotopes^20–22^. If the lamina counting method is coupled with two chronological tie points, such as the date that an actively growing sample is collected and the age of an artificial substrate on which a speleothem has grown, the chronological accuracy of the lamina counting method can be tested.

In this study, we exploit seasonal variations in trace elements to construct chronologies. Thus far, such methods have relied on one trace element with presumed annual cyclicity to construct a chronology^18, 23^. While the chosen element varies, it is predominantly bedrock-derived^17, 18, 24^ e.g., Mg, Sr, Ba and U. These ions are affected by seasonal changes in water-rock residence times and seasonal cave ventilation^14^, and processes like prior calcite precipitation (PCP)^14, 22^. However, the use of one element alone may not be reliable, as the process that causes a smoothly varying annual signal may be confounded by additional processes. For example, variations in Mg can be affected by both PCP and aerosol deposition of Mg from sea spray^5, 25^. However, if multiple trace elements are used and a process-based understanding of the variations within stalagmites are applied, then we may derive a more confident chronology.

Speleothems from regions with strong seasonality, such as mediterranean climates, are more likely to preserve annual geochemical information. For example, in southwest Australia, which is the focus of this study, seasonal water availability and in-cave processes have been shown to produce seasonality in dripwater composition^6, 14, 26^. The combination of seasonal water infiltration and cave ventilation can produce strong seasonal gradients in PCP^27^. For seasonal water infiltration, water interacts with the host rock for a longer time in dry periods, this can increase the concentration of dissolved solutes^14^, while seasonal fluctuation in surface-cave *p*CO_2_ gradients can induce seasonal fluctuations in PCP. Specifically, PCP is the term used to describe precipitation of calcite along the water flow path feeding a stalagmite^14^. The preferential loss of Ca over other ions during PCP results in higher dripwater ion/Ca ratios and ultimately higher ion/Ca ratios in the speleothem. PCP was identified by Treble *et al*.^18, 26^ as a key control on ion/Ca ratios such as Mg/Ca, Sr/Ca, Ba/Ca and U/Ca in southwest Australian dripwaters and stalagmite chemistry. As well as producing potential information regarding inter-annual recharge and *p*CO_2_ gradients, these annual cycles may be exploited to construct annually-resolved chronologies.

In this paper, we use multivariate trace element analysis - similar to that used to ‘fingerprint’ volcanic eruptions from Belize^28^ – and a semi-automated dating method - similar to Smith *et al*.^23^ – to build a chronology from the annual variations in trace elements from three stalagmites from three separate caves. The trace elements analysed included: 1) bedrock derived elements (Mg, Ba, Sr and U); 2) soil-derived metals typically mobilised as organo-metal complexes or soil bound colloids^29^ (P, Al, Fe, U, Cu and Zn); and 3) other possible elements that may be abundant due to post-fire processes (P and Al) and growth-rate^27^. Further, using the annually-resolved chronology, we examine the trace element and growth-rate data in one stalagmite and compare it to the known history of environmental change and instrumental climate during the 20^th^ century. Specifically, we examine the impact of fire, cyclones and a drying climate and highlight the applicability of using annual cycles to build chronologies in regions with a mediterranean climate.

## Methods

### Site and Samples

**Figure 1:**
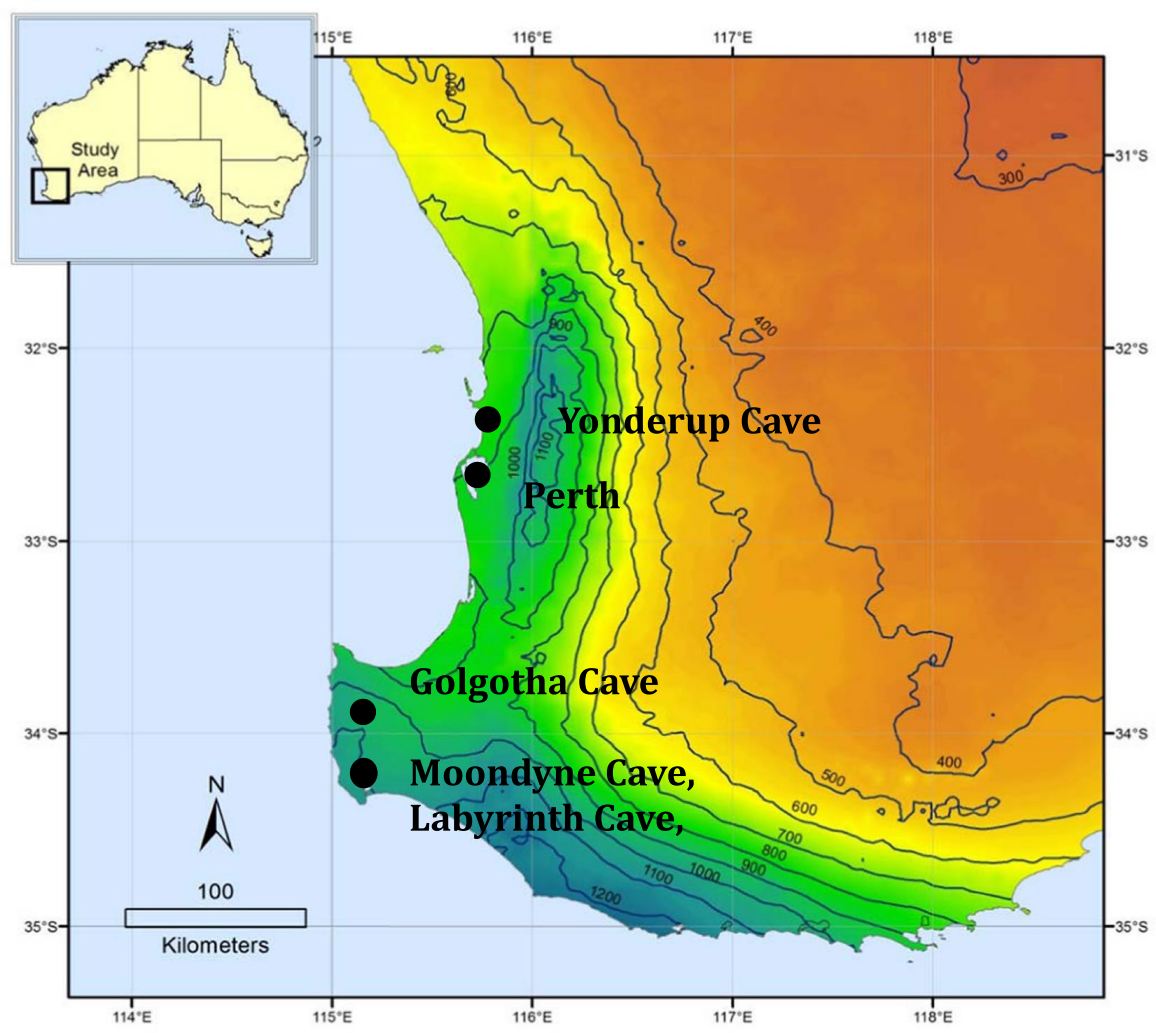
Geographic location of cave sites Yonderup Cave, Labyrinth, Moondyne Cave, and Golgotha Cave relative to Perth, and the regional rainfall isohyets in mm, with the mediterranean climate zone being between the coast line and the 600 mm isohyet line. Figure composed using ESRI ArcGIS v10.2 software (http://www.esri.com/software/arcgis/arcgis-for-desktop), and data based on Bureau of Meterology^30^ and Geoscience Australia material © Commonwealth of Australia (Geoscience Australia) 2016.

We used three stalagmites from different caves with varying depths in south-western Australia: Moondyne Cave (30 m deep), Yonderup Cave (4 m deep) and Labyrinth Cave (35 m deep). Details are summarised in Table 1. Moondyne and Labyrinth caves are approximately 300 m apart and lie within the Leeuwin Naturalist National Park, south of Perth, while Yonderup Cave lies in Yanchep National Park, north of Perth (Fig. 1). All caves are formed within the Tamala Limestone, a porous partially lithified calcareous dune sand. The karst formation process is “syngenetic”; as karstification occurs during lithification of the host rock^31^. Above Moondyne and Labyrinth caves is a Karri (*Eucalyptus diversicolor*) forest, and above Yonderup Cave is one of the last remaining Tuart (*Eucalyptus gomphocephala*) forests worldwide. Both forests have a dense understorey. The southwest region has a mediterranean climate with dry hot summers and colder wet winters and an average annual temperature of 15.1 °C. Moondyne Cave and Labyrinth Cave in the south receive an annual rainfall of 962 mm, while Yonderup Cave in the north receives an annual rainfall of 797 mm (Table 1).

**Table 1:** Summary of chronology, vegetation, climate and land-use and cave site characteristics for each region/cave site and stalagmite.

Stalagmite samples from the southwest region *(SWWA)*			
Location	Augusta, WA	Yanchep, WA	
Mean annual precipitation	962 mm (1900 to 2015) 912 mm (1970 to present)	797 mm (1900 to 2015) 698 mm (1970 to 2015)	
Mean annual temperature	17.0 °C (1999–2015)	18.3 °C (1996–2015)	
Mean annual cave temperature	15.9–16.5 °C^18^	17.6 ± 0.4 °C (annual range 17.1–18.2 °C)	
Vegetation	Karri (*Eucalyptus diversicolor)* forest with a dense understorey	Tuart forest (*Eucalyptus gomphocephala)* with a dense understorey.	
Fire and Land-use History	Record of land-use change and settlement and fires above the cave from 1958 to present.	Record of land-use change, development and fires in the park from 1960 to present.	
Cave Site	Labyrinth Cave 31.547°S, 115.69°E	Moondyne Cave 34.27°S, 115.098°E	Yonderup Cave 34.27°S, 115.098°E
Samples	LAB-S1 (all data unpublished)	MND-S1 (published data, Treble *et al*. 2003 and 2005)	YD-S2 (all data unpublished)
Trace elements	Mg, Sr, Na, U, Ba, P	Mg, Sr, Na, U, Ba, P	Mg, Sr, U, Ba, P, Pb, Cu, Al, Zn
Growth rate	0.35 mm	0.37 mm	0.07 mm
Cave depth (m)	35 m	30 m	4 m
Age span* (AD)	Unknown	1911 to 1991 AD	1832 AD (±54 years) to 2004 AD

Surface temperatures and rainfall data are from Cape Leeuwin (9518) and Gingin (9178) stations^30^. Cave temperature for Yonderup was calculated from 4 hourly measurements from July 2005 to November 2006. (*) Age span based on known age constraints for MND-S1 and U-Th dates for YD-S2. See supplementary Table 1 for more detail on U-Th dates.

The history of land-use change, such as disturbance to native vegetation by farming, fire (Supplementary Figure 2), road and tourism development, is known for all sites throughout the 20^th^ century based on records held by the Department of Parks and Wildlife in Western Australia. Complete records of daily rainfall are available from Gingin, ~30 km; and from Lower Chittering, ~40 km from Yonderup Cave for the whole 20^th^ century. The closest station (19 km), Wanneroo, has a temporally inconsistent record prior to 1980^30^. To distinguish dry and wet periods in climate a cumulative water balance (CWB) was calculated by applying a residual mass curve (RMC), following the methods of Hurst^32^, to monthly precipitation minus actual evapotranspiration (P – AET) data for the 20^th^ century. The RMC is the cumulative sum of the monthly anomaly from the pre-1970 (dry period) mean. This is used to generate a time series of cumulative potential water surplus or deficit starting from 1900, highlighting above average or below average P – AET.

Cave dripwater monitoring data is available for both regions from Yonderup Cave^6^ (2005–2011) and Golgotha Cave (2005–2015) 20 km from Labyrinth and Moondyne Caves^7^. These data include approximately monthly measurements of drip rate, dripwater solute chemistry and oxygen isotopes at both Yonderup and Golgotha Cave, as well as in-cave atmospheric measurements (temperature and *p*CO_2_) at Golgotha Cave. Stalagmite MND-S1 is from Moondyne Cave and grew on a cave tourist boardwalk and has been reported in detail in Treble *et al*.^18, 20, 33^. The boardwalk was in place 1911–1992, providing definitive constraints on our chronology. The Yonderup Cave stalagmite, YD-S2, was obtained from Site 1a in Nagra *et al*.^6^. YD-S2 is constrained by three U-Th age measurements, reported in this study, and the date of sampling. Stalagmite LAB-S1 is from Labyrinth Cave and its age is constrained by the date of sampling only, as the basal age was unable to be precisely constrained using U-Th owing to high initial detrital Th. LAB-S1 and YD-S2 stalagmites were dated at the University of Melbourne using U-Th mass spectrometry following the methods reported in Hellstrom^34^. We also note that LAB-S1 has cracks that account for approx. 2 mm of the sample length. We use the average growth-rate of 0.38 mm/year for the sample (based on the peak counting process in step 5) to fill in this gap when building the chronology.

### Trace element measurements

For trace element analyses, all samples were mounted in epoxy resin blocks and polished flat. The trace element data were acquired at the Australian National University (ANU), using excimer laser ablation inductively-coupled plasma mass spectrometry (ELA–ICPMS). The method is outlined in detail in Eggins *et al*.^35^ and Treble *et al*.^18^. The choice of elements measured in each speleothem was limited to those elements that could be detected in sufficient concentrations above background using the aperture required to obtain sub-seasonally resolved data. Aperture sizes were: 32 µm circular spot (MND-S1), 5 × 35 µm slit (YD-S2) and 20 × 40 µm slit (LAB-S1), with the wider axis of the slits perpendicular to the growth axis. Scan speeds ranged from 0.5–1.5 mm/min. Combined with a laser frequency of 15 Hz this produced data points approximately every 2–20 µm, depending on the growth rate of the sample. The data were reduced and standardised to NIST612 (Sr: 78.4, Ba: 41.0, Mg: 85.09, P: 39.1 and Na: 103 870 ppm) or NIST614 (Al: 10600, Cu: 1.19, Zn: 2.16, Pb: 2.07 and U: 0.8) glasses^36, 37^, as per the method in Treble *et al*.^18^. The data for MND-S1 were previously published in Treble *et al*.^18^. Numerous transects of data were acquired for each sample along and parallel to the central growth axis of the sample to reduce within stalagmite variability in our measurements^38^. Variability between adjacent transects was previously assessed in Treble *et al*.^18, 34^ for MND-S1 and is similar for the new data presented in this study for YD-S2 and LAB-S1. Three transects were used for the stalagmite chronologies in this study, with the exception of LAB-S1 for which only two transects had been acquired.

### Building annually-resolved chronologies for stalagmite datasets

Below we outline the steps taken to construct annually-resolved chronologies from trace element data by converting the annual cycle information from depth to time. We employed principal components analysis (PCA), a multi-dimensional correlation analysis method, using Originlab to identify the presumed common annual component among the trace elements. An automated peak counting program ‘Peak Analyser’ in Originlab was also used to locate peaks and their distance in each transect. For the latter, we adopted the Savitzky-Golay filter (as it best preserves peaks and troughs in these data) and the first derivative method options.

1. For each stalagmite, we first interpolated the data in each laser ablation transect to an equal number of points. The number of points was at least the minimum number of points in any of the transects, to minimise smoothing. This resulted in consistency between multiple transects within each stalagmite. Linear interpolation was considered to be unsuitable as it can inhibit preservation of natural non-linear variability. Instead, cubic-spline function options were used to preserve the maximum amount of variability between peaks and troughs within the series.
2. The concentration data for each element in each transect was normalised in order to better compare variability between different elements.
3. The data from each transect were concatenated into a single dataset for the PCA. This was done to minimise the influence of between transect or spatial variability on the PCA. We chose the common elements among the stalagmites which have been suggested in a previous study^18^ at our site to show near-annual variability (Mg, Sr, Ba and U) (experiment PCA A). We also investigated different suites of trace elements such as, Ba, Sr and U (PCA B). After the PCA, the concatenated principal components were split back into their respective transect compartments.
4. The automated peak counting method was applied to the PC1 from step 3, since PC1 explains the most variability for each transect. Since each stalagmite had a different growth-rate, a sampling window that reflects approximately one year of growth was chosen. This automated process required verification, which we detail further below.
5. The age-depth data from the peak counting output were used to generate a linear time series for each transect. The age-depth data were used also to calculate growth-rate, with one year of growth assumed to be the distance between two peaks.
6. The data from step 5 were resampled using the Savitsky-Golay filter, such that there were exactly 12 points per year, i.e. approximately monthly, under the assumption that growth-rate is near-constant year-round.
7. The transects are then averaged to produce a ‘master’ chronology.

The above method was also applied to the Sr data from each stalagmite (omitting step 3) to assess the accuracy of the method using a multi-element PCA approach versus a single element.

Treble *et al*.^18, 38^ pointed out that multiple transects of trace element measurements should be used when constructing a chronology from speleothem trace element data. This is to minimise uncertainty in the eventual chronology owing to poorly defined annual cycles in some transects, analogous to ‘missing’ or ‘multiple’ rings in dendrochronology^38^. To refine the automated peak counting process, we first identified ‘double peaks’ as follows: if two given peaks fell within one sampling window and showed troughs at less than 50% of the average peak height, these were identified as a double peak and eliminated. Second, if no peak was identified within a distance greater than 200% of the average growth-rate and there is variation of at least 30% of the average peak height, we assume there is a missing peak. Third, the identified peaks were cross-checked across the three transects for each stalagmite. In subsections of a transect, where the peaks in each PC1 were difficult to align, due to within stalagmite variability and low growth-rate i.e. YD-S2, clear recognisable common features or ‘tie-points’ i.e. large spikes, or cycles recognisable among all tracks were used to guide peak alignment.

## Results

**Figure 2:**
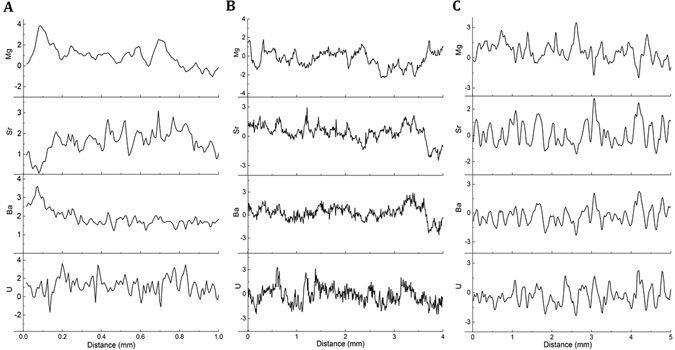
Typical sub-section of common trace element data (standardised) in each stalagmite (**A**) YD-S2, (**B**) LAB-S1 and (**C**) MND-S1. Note the x-axis varies for each stalagmite due to the different growth rates for each stalagmite; average growth rates are 0.41 mm (MND-S1), 0.065 mm (YD-S2), 0.352 mm (LAB-S1).

**Figure 3:**
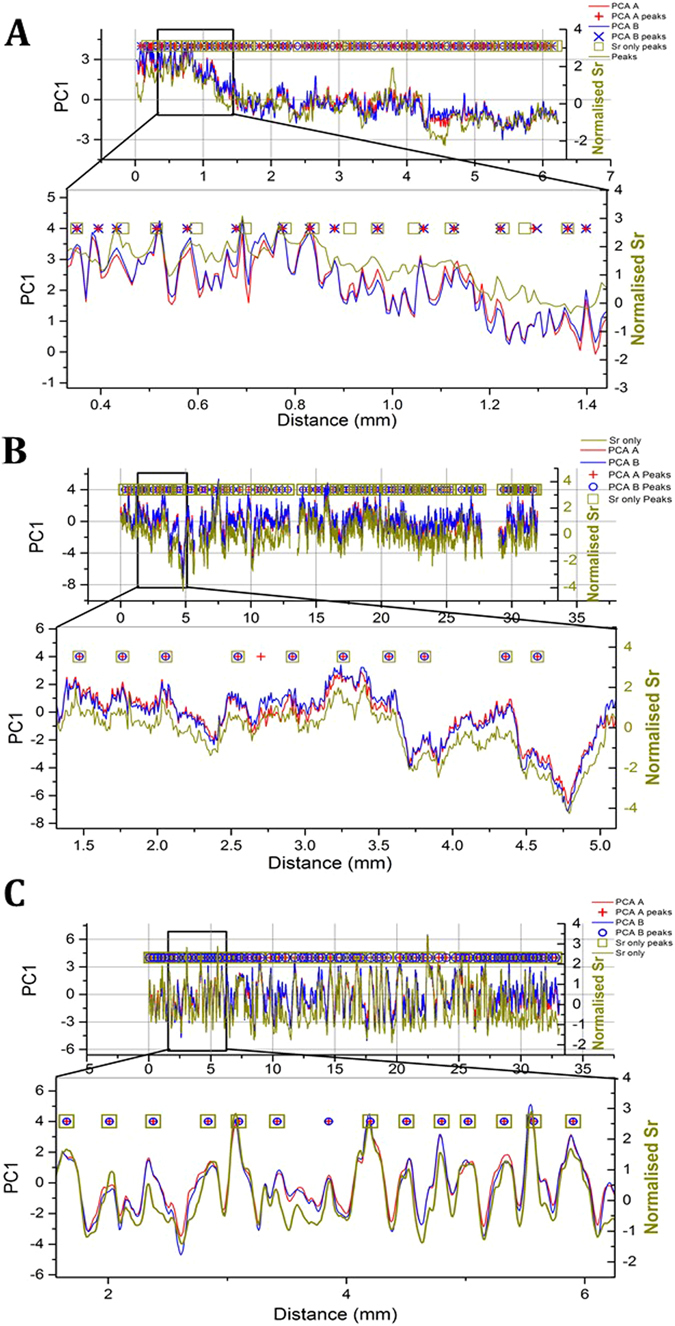
A comparison between peaks identified using three difference PCA experiments (PCA A, B and Sr only) for stalagmites YD-S2 (**A**), LAB-S1 (**B**), and MND-S1 (**C**). Only the PC1 time series output from one transect is shown for clarity. All methods locate similar, in phase peaks, although the output from Sr only tends to be smoother and have fewer peaks.

Figure 2 shows a portion of trace elements that were common in each stalagmite and have been shown to display annual cyclicity in other studies at our sites^6, 18^ (Mg, Sr, Ba and U). As stated, the PCA/peak counting method for building chronologies will be most effective if only those elements that have the clearest cycles are included, although it can be seen from Fig. 2 that the choice of elements may vary between stalagmites. For example, for MND-S1 (Fig. 2C) U, Ba and Sr show the clearest cyclicity while Mg is less clear. The results from PCA’s A, B and the Sr only method, are shown in Table 2 and the time series of the first principal components from these outputs are shown in Fig. 3. There is little difference between the number of peaks identified for each stalagmite using either the PCA A or PCA B experiment. For example, approx. 78 peaks, close to the actual age constraint of 80 years, were found using either experiment (Table 2). Similar agreement was found between results for stalagmites LAB-S1 and YD-S2. However, using Sr only consistently resulted in the identification of fewer peaks (4–7%) for all stalagmites (Table 2).

**Table 2:** Summary of the number of peaks identified in transects, the method used to detect the peaks, and the age constraints for each stalagmite.

	LAB-S1			MND-S1			YD-S2		
	PCA A	PCA B	Sr	PCA A	PCA B	Sr	PCA A	PCA B	Sr
Transect 1	81	81	78	78	78	73	187	187	175
Transect 2	79	79	76	77	77	76	186	186	175
Transect 3	X	X	X	78	78	76	186	186	167
Automation method used	1st Derivative (Savitzky-Golay filter used 30 pt window)			1st Derivative (Savitzky-Golay filter used 30 pt window)			1st Derivative (Savitzky-Golay filter used 10 pt window)		
Age Constraint	None			80 years (board walk stalagmite, see Treble *et al*. 2003 and 2005)			U-Th 183 ± 54 years		
Comments	This sample has cracks that take up approx. 2 mm. Using the average growth rate of each cycle in our peak fitting chronology, this makes up about 5 to 6 years of missing data.			T2 missing latest year (1991), T1 has extra cycle at 10.25 mm			Chronology falls within the age constraints of U-Th dates

Table 3 shows the loading of each element onto the first two PCs from the three PCA experiments. Only the first two PCs are listed, as PC3 typically explained less than 10% of the variance. For all stalagmites, the amount of variance explained by PC1 was highest for experiment PCA B i.e. when the suite of elements used was restricted to Ba, Sr and U only. Including Mg in PCA A resulted in weaker loadings onto PC1 for all three stalagmites. It also resulted in negative loadings for PC1 for both LAB-S1 and MND-S1, implying Mg is out of phase to Ba, Sr and U (PCA A; Table 3). This implies that Mg is driven by additional or different processes, violating the initial assumption that the annual cycles are driven by the same process. Thus, the results from experiment PCA B (Ba, Sr and U) are utilised to build the chronologies, constraining the method to the elements that will produce the most accurate chronology.

**Table 3:** Summary of the trace element loadings for all the PCA combinations used.

			*Sr*	*Ba*	*U*	*Mg*	*P*	*Al*	*Zn*	*Cu*	*Pb*	Variance
PCA A (Ba, Sr, Mg, U)	*YD-S2*	PC1	0.476	0.583	0.495	0.433						66%
		PC2	0.490	−0.217	0.451	−0.714						22%
	*LAB-S1*	PC1	0.595	0.489	0.463	−0.438						58%
		PC2	−0.009	0.478	0.291	0.829						19%
	*MND-S1*	PC1	0.496	0.547	0.501	−0.452						78%
		PC2	0.563	0.205	−0.061	0.798						12%
PCA B (Ba, Sr, U)	*YD-S2*	PC1	0.573	0.586	0.573							76%
		PC2	0.717	−0.019	−0.697							13%
	*LAB-S1*	PC1	0.628	0.571	0.528							67%
		PC2	−0.108	−0.608	0.786							22%
	*MND-S1*	PC1	0.570	0.607	0.554							86%
		PC2	−0.644	−0.090	0.760							12%
Local PCA C (YD-S2 only 20^th^C All trace elements)	*YD-S2*	PC1	−0.340	−0.259	−0.277	0.019	0.283	0.360	0.438	0.382	0.434	33%
		PC2	0.431	0.525	0.461	0.317	0.120	0.174	0.285	0.219	0.227	27%

PCA A and B are used in the chronology building section, while local PCA C is used for the interpretation of the soil vs. bedrock signal in the YD-S2 extreme events section.

**Figure 4:**
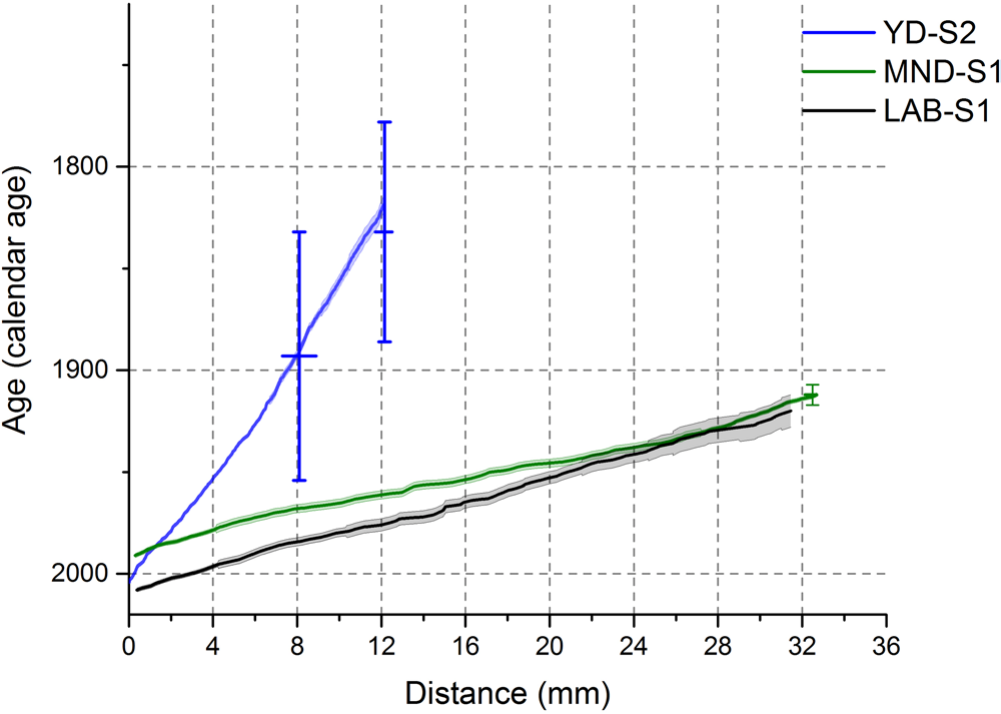
An age depth model for all samples, comparing our PCA B chronology vs. age constraints (U-Th dates for YD-S2, and substrate dates for MND-S1). The x-axis is distance below the top of the stalagmite. The age uncertainty for LAB-S1 is ±253 years; hence the basal age of this sample could not be constrained.

An age-depth plot comparing chronologies built from Ba, Sr and U peaks for all three stalagmites is shown in Fig. 4. Age constraints for MND-S1 and YD-S2 are also shown and U-Th dating results for stalagmites YD-S2 and LAB-S1 are presented in Supplementary Table 1. Judged against the exact age constraints for MND-S1, the annual-cycle based chronology yields a 2% uncertainty for MND-S1. For YD-S2, the basal date suggests that it is modern although the U-Th age uncertainties are large ±54 to 572 years; 2σ; depending on the estimated uncertainty for (^230^Th/^232^Th), hindering the comparison with the trace element chronology. Likewise, LAB-S1 has an U-Th age uncertainty that is also too large (±253 to ±754 years; Supplemental Table 1) to assess against the annual-cycle based chronology. Performing additional analyses is not likely to significantly improve the estimate of (^230^Th/^232^Th)_initial_.

Stalagmites YD-S2 and LAB-S1 are characterised by relatively low U content (~0.1 ppm) and low (^230^Th/^232^Th) (in the range 0.9 to 3.1) (Supplemental Table 1). Significant amounts of ^230^Th derived from detrital contamination at the time of speleothem formation is one of the major obstacles in producing precise U-Th chronologies for speleothems and it means that substantial corrections are needed for young speleothems in particular. A typical correction approach is the stratigraphical constraint method, where a range of (^230^Th/^232^Th)_initial_ is estimated under the condition that corrected ages are in stratigraphic order^39^. However, it was not practical to conduct multiple age measurements for these young speleothems and two alternative approaches were undertaken. Firstly an a *priori* estimated (^230^Th/^232^Th)_initial_ of 1.5 with a conservative uncertainty of ±1.5 were used. Second the U-Th corrected ages were matched to the peak fitted chronology to estimate the (^230^Th/^232^Th)_initial_. This involved correcting U-Th ages using different (^230^Th/^232^Th)_initial_ values until each corrected U-Th age was aligned with the peak fitted chronology. The best estimate of (^230^Th/^232^Th)_initial_ in this case is 1.33 ± 0.5 for YD-S2 and 0.9 ± 0.5 for LAB-S1 (Supplementary Table 1). All (^230^Th/^232^Th)_initial_ values used are within the uncertainty of a (^230^Th/^232^Th)_initial_ of ~0.9 ± 0.45 which is based on assumed averaged crustal U/Th ratio^39^. The resulting mid and basal ages for YD-S2 are 122 ± 61 and 183 ± 54 years before the date of sampling (2005) respectively, this strongly supports that it is a modern stalagmite, and that the variations in PC1 (PCA B) are also annual.

For LAB-S1, the calculated uncertainty is still too high (253 ± 754 years) to constrain the basal age of the sample. Performing additional analyses is not likely to significantly improve the estimate of (^230^Th/^232^Th)_initial_. The improved uncertainties are likely to still be too large, making the obtained date meaningless for any palaeoenvironmental study. This demonstrates the need for an alternative dating method for both modern and high-detrital Th stalagmites. The peak fitting method provides one avenue where annual cycles can be exploited to accurately and precisely date modern stalagmites.

## Discussion

### Annual variations from prior calcite precipitation (PCP)

With regards to cave dripwater for this region, Mg/Ca and Sr/Ca were positively correlated due to PCP^6, 26^ raising the expectation that they would be positively correlated in the stalagmites as well. This was the case for YD-S2, but not for LAB-S1 and MND-S1, for which Mg was negatively correlated with Ba, Sr and U. Temperature^15^ was previously eliminated by Treble *et al*.^18^ as a driver of annual Mg/Ca cycles in MND-S1, as the measured range in Mg/Ca is too large to be attributed to cave temperature variations. This argument also applies to LAB-S1 and YD-S2 here, since cave temperature ranges are similarly narrow (Table 1). Using a mass balance approach Treble *et al*.^7^ showed that whilst Sr was simply derived from the bedrock for the region, Mg was largely sourced from aerosol deposition due to coastal proximity and was also utilised as a biomass nutrient. Thus multiple factors may convolute the resulting Mg signal in stalagmites, hampering the use of this ion as a chronological tool.

In the case of YD-S2, a potential explanation for Mg being in phase with Ba, Sr and U is because PCP exerts a larger forcing at this site. This is supported by the mass of long soda-straw stalactites above our site, known as the ‘wheatfield’, which indicate a PCP dominated site^26^ (Supplementary Figure 1). Treble *et al*.^26^ showed that stalactite formation is enhanced at slowly dripping sites where drip rate is sufficiently slow for dripwater to equilibrate with cave air *p*CO_2_ and cause in-cave PCP. It is also noticeable that Yonderup Cave is much shallower (4 m) than Moondyne Cave or Labyrinth Cave (30–35 m). The shallow ceiling may enhance seasonal PCP as the volume of water stored above this site will be more vulnerable to seasonal infiltration and seasonal cave-air *p*CO_2_ fluctuations. Nevertheless, given the uncertainty around what is controlling Mg, and that including Mg in the PCA weakened the loadings on PC1, a combination of Ba, Sr and U arguably best reflect seasonal variation in PCP for the Yonderup Cave site as well.

Caves in climates with strong seasonality are more likely to have a seasonally-dominating PCP signal resulting in annual trace element cycles, due to the strong seasonal patterns in water availability and seasonal fluctuations in cave *p*CO_2_^26^. This expression of seasonality in speleothems is supported by a global model on seasonal cave ventilation, which found speleothems in mid to high latitude regions may be affected by any processes influenced by cave air CO_2_ concentrations including PCP^40^. Given the dominance of these seasonal processes in mediterranean climates, that exhibit strong seasonality defined by hot-dry summers and cold-wet winters, it is likely that this chronological method is also applicable to other water-limited regions around the world.

### Comparing the annually-resolved speleothem record to the historical record

**Figure 5:**
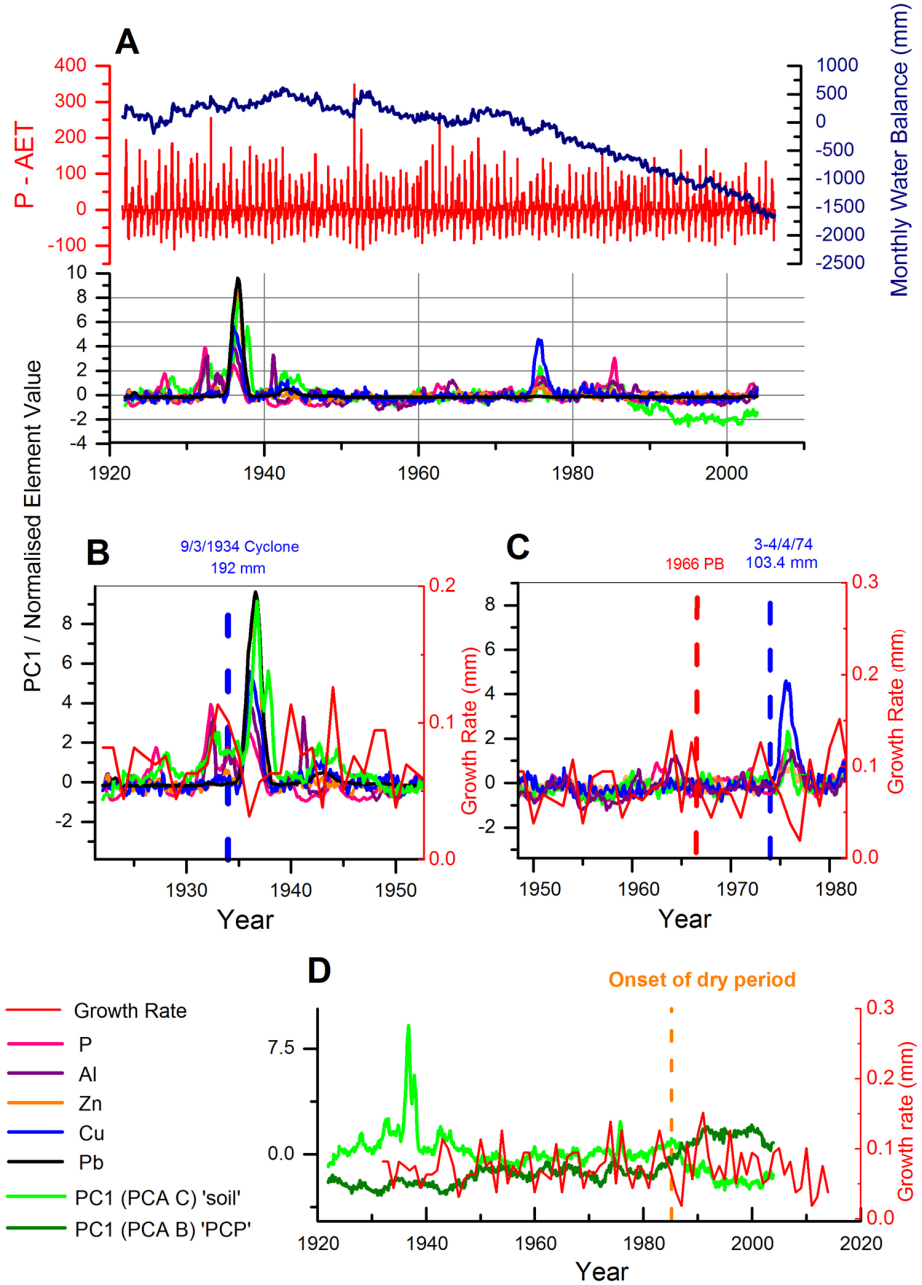
(**A**) A time series of water balance, normalised trace elements and PC1 (PCA C) ‘soil’ for stalagmite YD-S2; (**B,C**) Enlargements of PC1 ‘soil’, PC1 (PCA B) ‘PCP’, growth rate, and trace elements for 1934 cyclone and 1974 extreme rain events; and (D) PC1 ‘soil’, PC1 ‘PCP’ and growth rate with the period of post-1975 drying indicated. Trace element data are presented on separate panels in elementary Fig. 4.

Based on the PCP-based annual-cycle chronology built for YD-S2, the trace element, growth-rate and PCA data are compared to surface environmental changes over the 20^th^ C in Fig. 5. Yonderup Cave is a shallow cave and likely to be the more sensitive to surface environmental disturbances. It also has the most extensive historical record of land-use change, climate and fire. All elements measured in YD-S2 are included in PCA C i.e. P, Al, Cu, Zn and Pb also. In the case of PCA C, metals Sr, U and Ba that earlier were used to build the chronology, now load more strongly onto PC2 rather than PC1 (Table 3). In PCA C, metals Al, P, Pb, Cu, and Fe load more strongly onto PC1 and explain 33% of the trace element variability, while PC2 explains 27%. The sign of these loadings (Table 3) also indicate that they have anti-phase behaviour i.e. when PC1 is positive, metals Sr, U and Ba decrease while the other metals (Al, P, Pb, Cu, and Fe) increase.

Metals Al, Fe, Pb and Cu are all elements associated with organo-metal colloids from the soil that have been shown to enter caves in periods of water excess^29^. This is also true for P which has been shown to be a useful rainfall proxy^18^. Out of Sr, Ba and U, which were earlier attributed to a bedrock source, Sr has been confidently constrained to the bedrock^7^ for this region. An inverse relationship between P and Sr has previously been attributed to a soil vs. bedrock signal^15^. The PCA C analysis suggests that the soil-derived metals versus bedrock-derived metals behave in anti-phase (PC1). However, it needs to be determined whether PC1 is driven by climatic or local changes. To explore this, we focus on four features in the dataset over the 20^th^ century.

First, the record of land-use change and development that occurred within 2 km of Yonderup Cave in the early 20^th^C. Development of the Yanchep National Park for tourists began in the early 1930’s, peaked in the mid-1930’s and declined after 1939 due to the outbreak of World War II^41^. This included the development of new roads to accommodate a heavy influx of cars and buses carrying tourists into the park and the planting of up to 10,500 trees by 1939; a dramatic change in the local environment^41^. During this period, multiple abrupt peaks occur in P and Al (Fig. 5B), as well as a large synchronous peak in Cu, Pb and Zn. It is likely that land-use changes are responsible for the peaks in these elements although direct attribution is difficult as details of what occurred directly above the cave is not known.

Second, we consider the impact of fire (Fig. 5C). Only one recorded fire went directly over the surface of the cave; a prescribed burn in 1966, although little to no significant trace element response is recorded. This is likely due to the known low intensity of this prescribed burn in comparison to a wildfire. There is however, a negative excursion in growth-rate, which has been postulated as an effect of fire on stalagmite composition^6^, although it is constrained to a single data point. Hence, it is difficult to interpret any fire-related effects from this low-intensity burn. Further, our suite of elements did not include those postulated previously^6^ to increase post-fire: S and K, thus further research needs to be done on these elements in modern sites with a fire record.

Third, a well-known climatic shift towards drier conditions^20, 33^ occurred around 1975 resulting in a clear decline in the water balance (Fig. 5A). In the post-1975 dry period, there is a long-term rise in the bedrock-dominated signal (PC1 from PCA B) and a long-term decline in the soil-dominated signal (PC1 from PCA C; Fig. 5D). The rise in the bedrock-dominated signal is linked to an increase in Ba, U and Sr post-1975 (Supplementary Figure 1). Given that these are bedrock sourced, it suggests an increase in water-rock residence time or PCP. Stalagmite growth-rate simultaneously declines during the dry period, consistent with reduced dripwater Ca concentrations owing to increased PCP.

Finally, there is a transitory peak in soil-derived elements around 1975–76 in the YD-S2 record, coinciding with lower growth rate, which is not related to the persistent drying trend. This aligns, within chronological uncertainty, with heavy rainfall (90.2 mm) recorded at Gingin station on April 3^rd^ 1974. Given the potential link, we also consider extreme rainfall events that occurred near this site at other times in the 20^th^C. There are six days in total of rain above 90 mm (extreme rainfall exceeding the 99^th^ decile in daily rainfall) in this region^30^ recorded for the 20^th^C (Supplementary Figure 3 and Supplementary Table 3), but only two exceed 90 mm at more than one site, March 9^th^ 1934 (a tropical cyclone; http://www.bom.gov.au/cyclone/history/wa/perth.shtml) and October 6^th^ 1945. In the stalagmite record, there is a clear peak in the soil-derived PC1 signal in 1935 (falling within 1% chronological uncertainty of the recorded cyclone) dominated by peaks in Cu, Pb, Zn, Al and P. This cyclone brought heavy rainfall (192.3 mm) at both Gingin and (116.3 mm) Lower Chittering stations. However, no peak was recorded in 1945 in response to heavy rainfall recorded at both Gingin and Lower Chittering (97 mm).

The metals Al, Pb, Cu and Zn in the speleothems are all mobilised from the soil as organo-metal complexes or linked with colloids, which can be either flushed into the cave from extreme rainfall or dispersed as aerosols into the cave system^29, 42^. A simultaneous rapid decline in growth-rate during this colloidal peak would suggest this is a result of rapid infiltration. Growth-rate is controlled by the concentration of calcium dissolved in dripwaters, and this would decrease rapidly in high infiltration events, due to dilution. Therefore, it is likely that the peaks in metals observed in 1935 and 1975 are both due to heavy rainfall. This raises the prospects of reconstructing paleotempestological records using trace elements from speleothems.

## Conclusions

We have presented a new method to utilise high-resolution trace element data, in particular Ba, Sr and U, to form annually-resoalved chronologies for stalagmites. The annual variation in the stalagmites presented is likely due to seasonal variations in prior calcite precipitation (PCP) from seasonal infiltration and/or seasonal fluctuations in cave *p*CO_2_. We demonstrated that chronologies with an uncertainty of ~2% can be achieved. This is of similar precision achieved for tree ring-based chronologies (~1–4%)^43^ and ice core layers (~4%^44^). We also argue that this is due to the strong seasonality in mediterranean climate environments. Therefore, this method could be applied to caves elsewhere in other mediterranean climate regions such as, Italy, Spain, Greece, Oman, central Chile, western California, southern Australia and the south-western cape of South Africa, to build annually-resolved speleothem chronologies.

Based on this method, we construct a new speleothem record for the 20^th^ C for this region. Extreme rainfall from a recorded cyclone in 1934 is recorded within 1% chronological uncertainty, via a peak in soil-derived trace elements. This demonstrates the potential of soil-derived trace elements as proxies to build paleo-records of extreme rainfall in mediterranean regions. A clear long-term drying trend is also recorded in PCP-affected elements (Ba, Sr and U) and in speleothem growth-rate in response to the post-1975 rainfall decrease, similar to findings for another speleothem record (MND-S1) reported earlier^18^. Our findings further demonstrate and strengthen the potential for using stalagmite-based records for climate reconstruction in the southwest Australian region, as well as other regions with a mediterranean climate.

## Electronic supplementary material

Supplementary Figures and Tables

Supplementary Dataset
