# Supplementary material for: Dating stalagmites in mediterranean climates using annual trace element cycles
Source: Sci Rep. 2017 Apr 4;7:621. doi: 10.1038/s41598-017-00474-4 (PMC5428411; doi:10.1038/s41598-017-00474-4)
Supplement: Supplementary file 1 — Supplementary Figures and Tables [file 41598_2017_474_MOESM1_ESM.doc]

## Supplementary Information:

## Dating stalagmites in mediterranean climates using annual trace element cycles

## Gurinder Nagra^1*^, Pauline C. Treble^1, 2^, Martin S. Andersen^1^, Petra Bajo^3^, John Hellstrom^3^, Andy Baker^1^

*^1^ Connected Waters Initiative Research Centre, University of New South Wales, Sydney, NSW, 2052, Australia.*

*^2^ Australian Nuclear Science and Technological Organization, Lucas Heights, NSW, 2234, Australia.*

*^3^ School of Earth Sciences, University of Melbourne, Melbourne, VIC, 3010, Australia.*

| Sample name | Mass (mg) | U  (ppb) | Depth from top  (mm) | (^230^Th/^238^U)  (± 95% unc) | (^234^U/^238^U)  (± 95% unc) | (^232^Th/^238^U) (± 95% unc) | (^230^Th/^232^Th) | (^230^Th/^232^Th)_i_  (± 95% unc)  “A priori” estimated | Age  (±2σ)  (ka) | (^234^U/^238^U)_i_ (± 95% unc) | (^230^Th/^232^Th)_i_  (± 95% unc)  Estimated by adjustments to peak fitting chronology | Age  (±2σ)  (ka) |
| --- | --- | --- | --- | --- | --- | --- | --- | --- | --- | --- | --- | --- |
|  |  |  |  |  |  |  |  |  |  |  |  |  |
| YDS2 UM02 | 45 | 81 | 4.35 (0.75) | 0.0039 (0.0003) | 1.1075 (0.0058) | 0.00385 (0.00002) | 1.0 | 1.50  (1.5) | -0.192 (0.572) | 1.1074 (0.0058) | 1.33  (0.5) | -0.129 (0.191) |
| YDS2 UM03 | 122 | 93 | 8.10 (0.80) | 0.0029 (0.0002) | 1.1129 (0.0039) | 0.00117 (0.00001) | 2.4 |  | 0.102 (0.174) | 1.1129 (0.0039) |  | 0.122 (0.061) |
| YDS2 UM01 | 187 | 105 | 12.15 (1.05) | 0.0034  (0.0001) | 1.1118 (0.0040) | 0.00108 (0.00001) | 3.1 |  | 0.165 (0.159) | 1.1119 (0.0040) |  | 0.183 (0.054) |
| LABS1 UM05 | 137 | 119 | 31.60 (0.84) | 0.0066  (0.0003) | 1.0501 (0.0022) | 0.00481  (0.00003) | 1.4 | 1.50  (1.5) | -0.069 (0.754) | 1.0501 (0.0022) | 0.9  (0.5) | 0.233  (0.253) |

**Supplementary Table 1.** U-Th results for YDS2 and LABS1. Isotopic ratios are activity ratios and uncertainties are expressed in brackets as 95% confidence intervals. All ages are reported as ka before present, where present is the date of sampling, 2005.

|  | **P** | **Mg** | **Al** | **Sr** | **Ba** | **U** | **Na** | **Zn** | **Cu** | **Fe** | **Pb** |
| --- | --- | --- | --- | --- | --- | --- | --- | --- | --- | --- | --- |
| **YDS2** | | | | | | | | | | | |
| **Mean** | 0.0160 | 8.3185 | 0.0246 | 0.3911 | 0.0093 | 0.0000 |  | 0.0021 | 0.0002 | 0.0908 | 0.0001 |
| **St. dev.** | 0.0221 | 3.0336 | 0.0066 | 0.0354 | 0.0028 | 0.0000 |  | 0.0021 | 0.0014 | 0.0139 | 0.0014 |
| **LABS1** | | | | | | | | | | | |
| **Mean** | 0.3092 | 6.6641 | 0.0908 | 0.0692 | 0.0011 | 0.0000 | 0.6770 |  |  |  |  |
| **St. dev.** | 2.1368 | 1.8361 | 1.4324 | 0.0608 | 0.0007 | 0.0001 | 3.0147 |  |  |  |  |
| **MNDS1** | | | | | | | | | | | |
| **Mean** | 0.1852 | 16.2400 |  | 0.1461 | 0.0081 | 0.0001 | 0.7961 |  |  |  |  |
| **St. dev.** | 0.0814 | 2.5069 |  | 0.0320 | 0.0014 | 0.0000 | 0.1623 |  |  |  |  |

**Supplementary table 2.** Summary of average concentrations of trace elements in each sample, shown in mmol/mol.


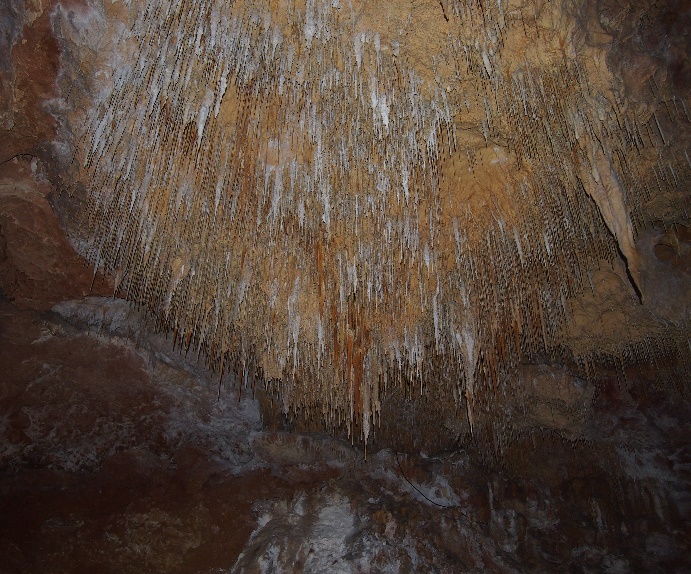


**A)**

**C)**

**Supplementary Figure 1.** Photo of the ‘Wheatfield’ of soda straw stalactites above YDS2; a key characteristic of in-cave PCP.


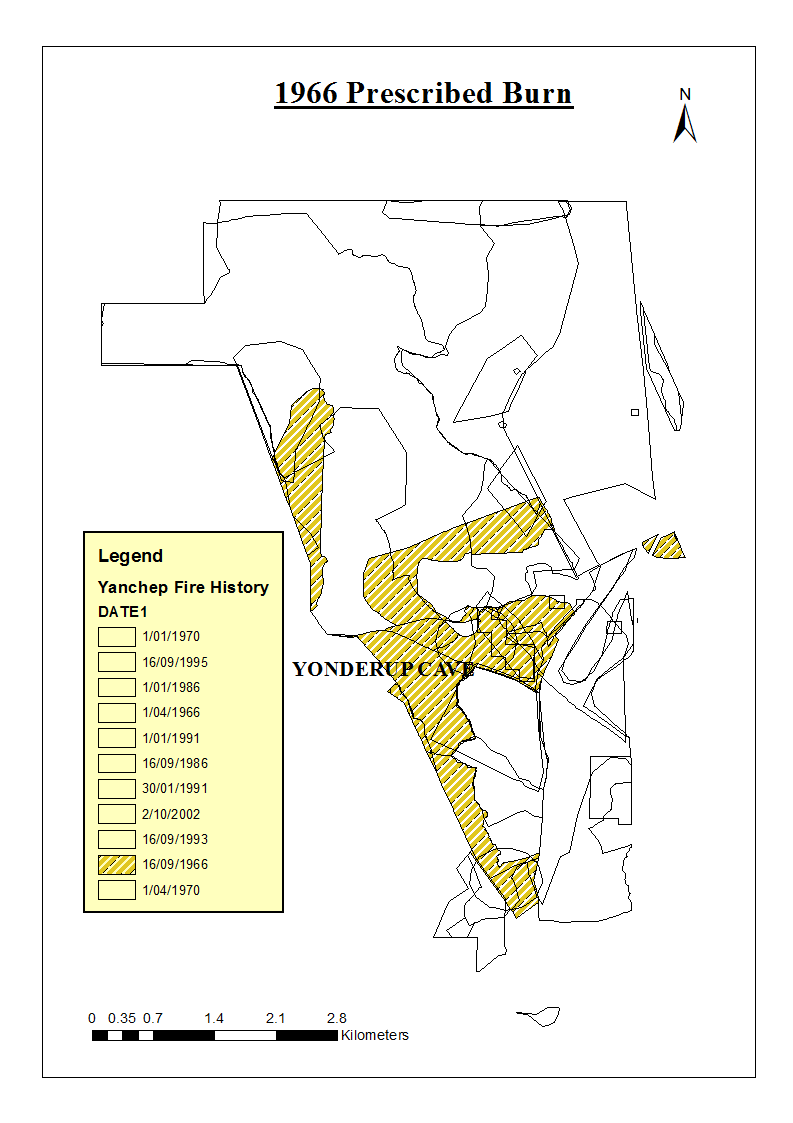
**Supplementary Figure 2.** The geographic distribution of the prescribed burn in 1966 in Yanchep National Park and the location of Yonderup cave; one of our cave sites. Figure composed using ESRI ArcGIS v10.2 software (http://www.esri.com/software/arcgis/arcgis-for-desktop).


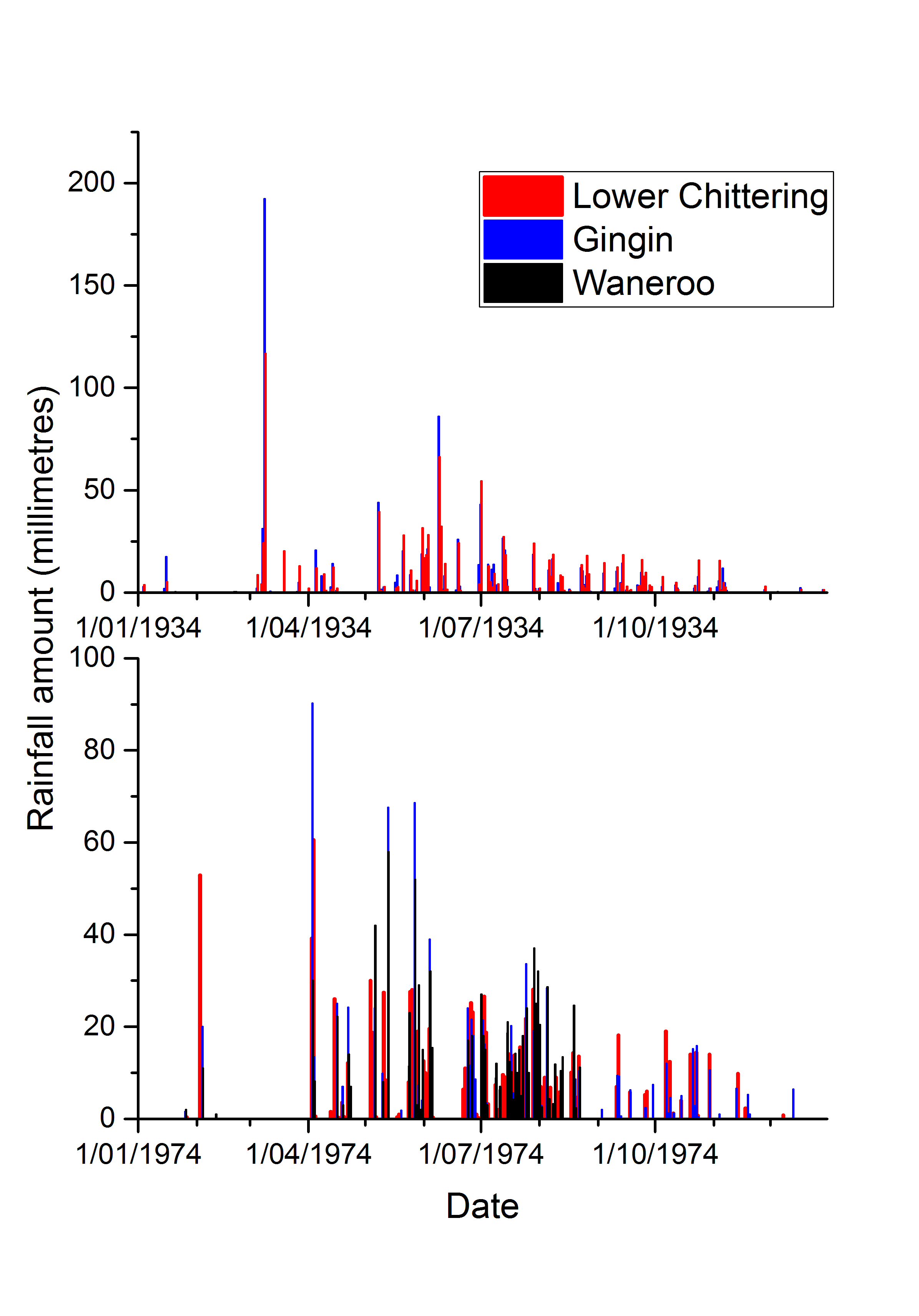


**A**

**B**

**Supplementary Figure 3.** Daily rainfall data for the years with the most extreme rainfall events 1934 (A) and 1974 (B).

|  | **Bureau of Meteorology station number** | **Year** | **Month** | **Day** | **Rainfall amount (millimetres)** | **Time period**  **( # of days)** | **Quality** |
| --- | --- | --- | --- | --- | --- | --- | --- |
| **Gingin** | 9018 | 1934 | 3 | 9 | 192.3 | 1 | Y |
|  | 9018 | 1945 | 6 | 10 | 116.3 | 1 | Y |
|  | 9018 | 1999 | 1 | 23 | 95.6 | 1 | Y |
|  | 9018 | 1974 | 4 | 3 | 90.2 | 1 | Y |
| **Lower Chittering** | 9009 | 1990 | 1 | 29 | 119.2 | 1 | Y |
|  | 9009 | 1934 | 3 | 9 | 116.8 | 1 | Y |
|  | 9009 | 1945 | 6 | 10 | 97 | 1 | Y |
| **Wanneroo** | 9105 | 1987 | 7 | 29 | 92 | 1 | Y |

**Supplementary Table 3.** Wettest days above 90mm closest to Yonderup Cave.


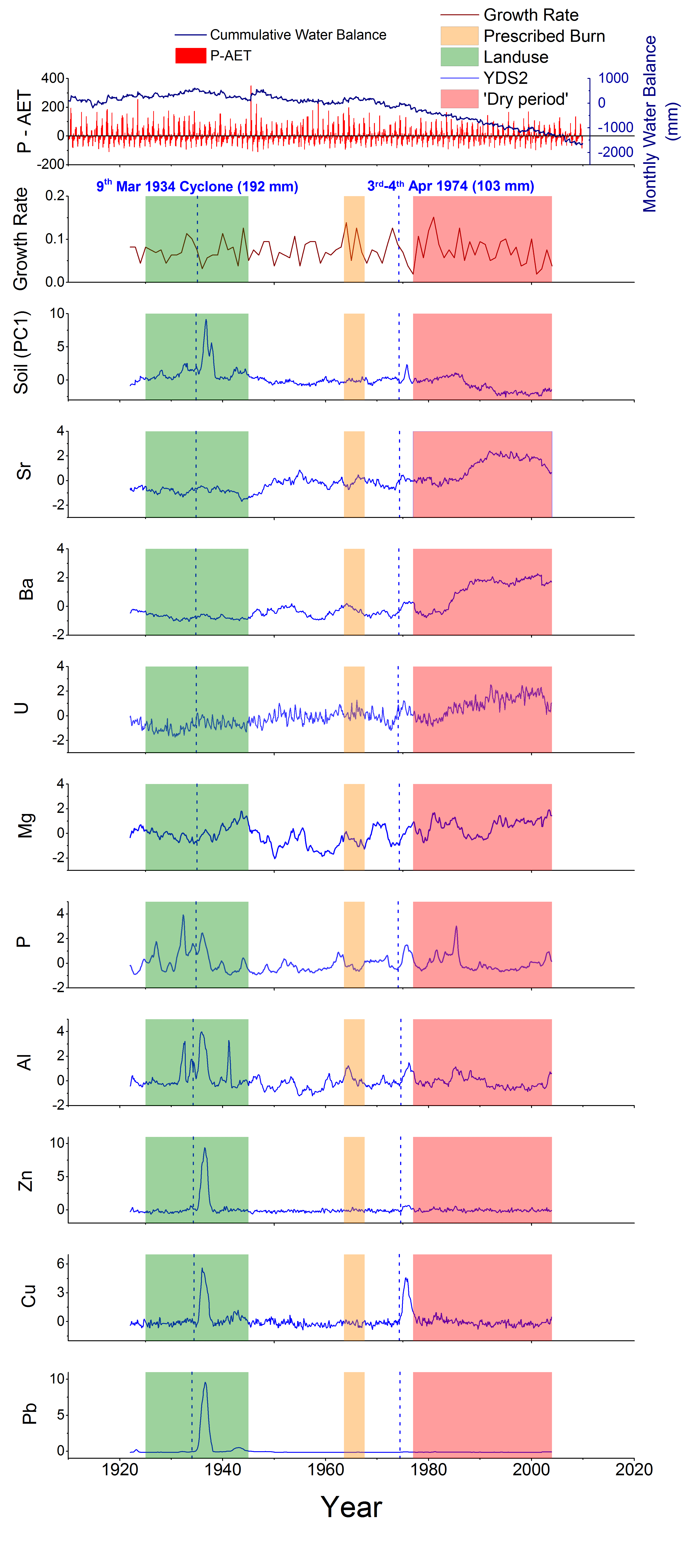


Supplementary Figure 4. Time series of water balance, speleothem growth rate, PC1 from PCA C ‘soil’ and normalised trace elements. Trace element concentrations are given in Supplementary Table 2. Intervals of known landuse change, fire and drying are shaded; and extreme rainfall events marked by dotted lines.
